# Recent Advances in General Game Playing

**DOI:** 10.1155/2015/986262

**Published:** 2015-08-24

**Authors:** Maciej Świechowski, HyunSoo Park, Jacek Mańdziuk, Kyung-Joong Kim

**Affiliations:** ^1^Systems Research Institute, Polish Academy of Sciences, Ulica Newelska 6, 01-447 Warsaw, Poland; ^2^Department of Computer Science and Engineering, Sejong University, Seoul, Republic of Korea; ^3^Faculty of Mathematics and Information Science, Warsaw University of Technology, Ulica Koszykowa 75, 00-662 Warsaw, Poland

## Abstract

The goal of General Game Playing (GGP) has been to develop computer programs that can perform well across various game types. It is natural for human game players to transfer knowledge from games they already know how to play to other similar games. GGP research attempts to design systems that work well across different game types, including unknown new games. In this review, we present a survey of recent advances (2011 to 2014) in GGP for both traditional games and video games. It is notable that research on GGP has been expanding into modern video games. Monte-Carlo Tree Search and its enhancements have been the most influential techniques in GGP for both research domains. Additionally, international competitions have become important events that promote and increase GGP research. Recently, a video GGP competition was launched. In this survey, we review recent progress in the most challenging research areas of Artificial Intelligence (AI) related to universal game playing.

## 1. Introduction

Games have always been an important platform for research on Artificial Intelligence (AI). Since the early days of AI, many popular board games, such as chess and checkers, have been used to demonstrate the potential of emerging AI techniques to solve combinatorial problems. Recently, some board games were declared nearly or completely solved (i.e., there are programs capable of playing a particular game optimally and neither humans nor other computer programs can perform better) [1, 2]. These programs are based on sophisticated tree-based search algorithms with well-designed evaluation functions, huge databases of game situations, and specially designed hardware chips. Although these programs have managed to reach world champion-level performance, it remains questionable whether they can match human-level game playing capabilities. In any event, the expansion from traditional board games to other types of complex games will continue to advance research on game AI problems.

Some research stresses the importance of human-style game playing instead of simply unbeatable performance [3]. For example, given a certain board configuration, human players usually do not check as many possible scenarios as computer players. However, human players are good at capturing patterns in very complex games, such as go [4] or chess [5, 6]. Generally, the automatic detection of meaningful shapes on boards is essential to successfully play large-branching factor games. The use of computational intelligence algorithms to filter out irrelevant paths at an early stage of the search process is an important and challenging research area. Finally, current research trends are attempting to imitate the human learning process in game play.

General Game Playing (GGP) was introduced to design game-playing systems with applicability to more than one specific game [7]. Traditionally, it is assumed that game AI programs need to play extremely well on a target game without consideration for the AI's General Game Playing ability. As a result, a world-champion level chess program, such as Deep Blue, has no idea how to play checkers or even a board game that only slightly differs from chess. This is quite opposite to humans' game-playing mechanism, which easily adapts to various types of games based on learning the rules and playing experience. In the context of GGP, the goal of an AI program is not to perfectly solve one game but to perform well on a variety of different types of games, including games that were previously unknown. Such an approach requires a completely different research approach, which, in turn, leads to new types of competitions and general-purpose algorithms.

Unlike game-specific AI research, GGP assumes that the AI program is not tightly coupled to a game and, therefore, requires formal descriptions of games similar to game manuals for human players. The formal description of such games is Game Definition Language (GDL) [8]. It is a text-based and logic-based description of game rules that can be used to model a diverse array of games ranging from those as simple as Tic-Tac-Toe to those as complex as chess. GGP programs must be able to parse and understand the GDL file of a given game. Using the GDL, it is possible to define new games by slightly changing the widely used common rules. This enables the definition of many games, a characteristic of GDL that is essential for measuring the performance of GGP programs. The use of GDL has become influential in GGP research through the introduction of the GGP competition. It has also led to a new definition of its video game extension, Video Game Description Language (VGDL).

Traditionally, GGP has focused primarily on two-dimensional board games inspired by chess or checkers, although several new approaches for General Video Game Playing (GVGP) have been recently introduced to expand the territory of GGP [9]. The goal of GVGP research is to develop computer algorithms that perform well across different types of video games. Compared with board games, video games are characterized by uncertainty, continuous game and action space, occasional real-time properties, and complex gaming rules. Researchers involved in GVGP are beginning to define their language, VGDL, which is equivalent to GDL in GGP research [10]. Additionally, GVDL comes with a new type of competition [11]. This is a new field of research that bridges video game AI research and traditional GGP research.

Since its introduction, GGP research has continued to progress. The AAAI GGP competition has provided an internationally acceptable venue for evaluating algorithms applied to GGP [12]. Based on the results of the competition, the progress in this research domain can be measured, and many promising techniques have emerged from the competition. Specifically, the use of the Monte-Carlo Tree Search (MCTS) has been widely adopted in GGP research [13]. Recently, GGP has expanded to other video games, including grid-style two-dimensional video games and Atari video games. For this review, we will focus on advances in GGP research since 2011.

This paper is organized as follows.  Section 2 describes advances in the MCTS method, the state-of-the-art GGP approach, with particular focus on Monte-Carlo (MC) simulation control mechanisms. The MCTS algorithm is very well suited to the GGP domain due to its general applicability because various games can be encountered during GGP tournaments. However, the main disadvantage of MCTS is that it makes very limited use of game-related knowledge, which may be inferred from a game description.  Section 3 addresses methods that are rooted in AI and take advantage of game-specific information. In Section 4, recent advances in game rules representations and parallelization of MCTS, both of which are critical aspects of building efficient tournament-level players are described. In Section 5, the use of GGP techniques for video games is introduced and promising research platforms are discussed.  Section 6 reviews the acceleration of GGP research through international competitions. Finally, the paper is concluded with a discussion about challenges and future directions.

## 2. GGP-Related Advances in MCTS

### 2.1. MCTS Overview

**Figure 1:**
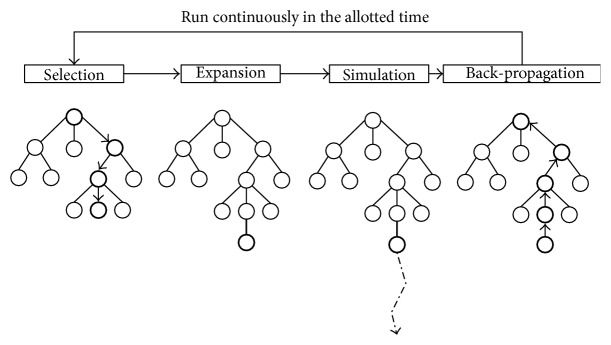
Four steps of the Monte-Carlo Tree Search algorithm.

Monte-Carlo Tree Search (MCTS) is the algorithm of choice by the most competitive General Game Playing agents. For a survey about the MCTS, please consult [13]. The authors of the survey aimed to embed an exhaustive knowledge about the algorithm including origins, mathematical foundations, the structure of the method, and numerous enhancements. A simple description of how the MCTS is applied by GGP players can be found in [14] for a player named Gamer. The algorithm iteratively searches the game tree starting from the current state in series of iterations until the allotted time runs out. An iteration consists of the following four steps: selection, expansion, simulation, and back-propagation depicted in Figure 1.

(1) *Selection Step*. The algorithm starts from a root of the game-tree and chooses a node within an already built part of the tree based on the nodes' statistics. Actions, which have been performing better so far, are tested more frequently. Typically, some kind of a confidence algorithm such as Upper Confidence Bounds applied for Trees (UCT) is used as shown in (1). The UCT algorithm is an extension to the flat Upper Confidence Bounds (UCB). Consider
  (1) $$\begin{array}{c} a^{*}=\arg\underset{a\in A\left(s\right)}{\max}\left\{\;Q\left(\;s,a\;\right)+C\sqrt{\frac{\ln\left\lfloor N\left(s\right)\right\rfloor }{N\left(s,a\right)}}\;\right\}, \end{array}$$
  where *s* is the current state, *a* is an action in this state, *A*(*s*) is a set of actions available in state *s*, *Q*(*s*, *a*) is an assessment of performing action *a* in state *s*, *N*(*s*) is a number of previous visits of state *s*, *N*(*s*, *a*) is a number of times an action *a* has been sampled in state *s*, and *C* is the exploration ratio constant.
(2) *Expansion Step*. It means extending the tree by a new node with the first unvisited state so far, that is, the first state found after leaving the tree.
(3) *Simulation Step*. After leaving the stored fragment of the tree, a random simulation is performed until a game termination is reached.
(4) *Back-Propagation Step*. The scores obtained by all players in the ended game are fetched and back-propagated (back-propagation) to all nodes visited in the selection and expansion steps.

Because the origins of Monte-Carlo methods are in statistical physics and for the UCT selection algorithm in optimization of a multiarm bandit payoff (gambling math), the success of this approach in games has been surprising. There have been significant amount of publications in the area of MCTS, but in this section we will focus only on papers related to the GGP. The main reason why this method has been so successful in a domain of universal game-playing programs is that it does not require any game-specific knowledge such as heuristic evaluation function for the assessment of position. The only requirement is to be able to simulate a game and read the results. Moreover, the MCTS is an anytime algorithm that can be stopped at any time and return the best move so far. It parallelizes and scales well as opposed to alpha-beta-like methods, which provide only linear improvement with exponential growth of the tree. A link between game-tree properties and performance of the MCTS can be found in [15]. The authors analyze such properties as follows:

- *Branching factor*: the average number of possible moves in a state impacting the tree width.
- *Tree depth*: connected to the average length of a simulation from the beginning of a game to the end.
- *Progression towards a natural termination*: each move that naturally brings the state closer to a terminal one. Examples of naturally progressive games given in the paper are* Connect 4*,* Othello*, and* Quarto*. Often, a natural termination is featured in games, where players fill a board and pieces once placed do not disappear; therefore the board eventually fills up completely. On the other hand, games without a natural termination often could go infinitely long without an artificial termination condition such as a maximum number of steps or a maximum number of state repetitions. Examples of such games are* Chess*,* Skirmish*, and* Bomberman*.
- *Existence of optimistic moves*: moves which are immediately good, that is, win a game or give a good result for the player in a few number of steps, provided that the opponent does not see a proper response (thus optimistic). However, if the opponent makes the right response, it usually puts him ahead of the player who made the optimistic move. Optimistic moves usually exist in games, where it takes lots of simulations to calculate the response compared to finding the good moves.

It was found out in [15] that comparing branching factor versus tree depth, there is no factor out of these two which influences the MCTS performance more. It depends more on the actual rules of a game being played. Both the larger branching factor and the deeper tree slow down the process and render the MCTS assessment less accurate. Progression towards a natural termination increases whereas existence of optimistic moves decreases the performance of the MCTS.

### 2.2. Reducing the Combinatorial Complexity

The UCB algorithm was designed to work with bandits giving payoff stochastically according to some unknown distribution. The payoff function is continuous within certain bounds. In GDL-I, while there is still randomness by means of uncertainty of what actions the other players will chose, the games are deterministic by structure.

#### 2.2.1. Sufficiency Threshold

In [16], the authors propose two optimizations known as* moving average return function* and* sufficiency threshold* to exploit the nature of games in GGP which are characterized determinism and a fixed number of scores available in every game. The general idea is to allocate more simulations to actions which evaluation is not clearly converging to a score defined in the game. A second idea is presented to distinguish between two similarly evaluated best moves. In such case, it is beneficial to allocate the whole budget to just one of these two moves. If the estimation stays high or even increases, then the move should be played. Otherwise, the second, not well-simulated one, should be played. The* sufficiency threshold* has been introduced by the same authors both in [16, 17] to optimize the allocation of simulations and tackle the issue of liability of the MCTS technique to choosing optimistic moves. It is defined as a parameter *α*, which affects the exploration parameter *C* in the UCT formula introduced in (1):

(2) $$\begin{array}{c} \hat{C}=\left\{\begin{array}{cc} C, & \text{when}\;\text{all}\;Q\left(s,a\right)\leq \alpha ,\\ 0, & \text{when}\;\text{any}\;Q\left(s,a\right)>\alpha . \end{array}\right. \end{array}$$

#### 2.2.2. Move Average Return Function

Move Average Return Function, introduced in [16], increases importance of newer simulation results as performed with more information and being more accurate of how the match can unfold. Let *r* denote the result from a simulation and *λ* be inverse to the number of simulations for small number of simulations and an arbitrary constant after reaching that threshold. The score update function becomes

(3) $$\begin{array}{c} Q\left(\;s,a\;\right)=Q_{\text{old}}\left(\;s,a\;\right)+\lambda \left(\;r-Q_{\text{old}}\left(\;s,a\;\right)\;\right). \end{array}$$

#### 2.2.3. Early Cutoff

The paper [18] brings two new extensions to the MCTS. The first one is applied in the simulation step and it is called* early cutoff*. The idea is to terminate a simulation earlier, as opposed to running it till the end, in order to save computation time and perform more simulations. The cutoff is based on two conditions: the depth from the starting state and goal stability. The goal is stable if it can be computed in nonterminal states, changes with low variance, and is correlated with the situation in the game. The notion of the goal stability was borrowed from earlier papers in GGP [19]. The pseudo-code for the early cutoff is as in Algorithm 1.

#### 2.2.4. Unexplored Action Urgency

The second one is* Unexplored Action Urgency* in which there is no longer a requirement to select each action at least once. An urgency of an action is defined as follows:

(4) $$\begin{array}{c} \text{urgency}=50+C\sqrt{\ln N\left(s\right)}*\text{discount}, \end{array}$$

where *N*(*s*) is the number of visits to a state and discount is the number of unexplored actions divided by the number of total actions available in the state.

Now if any action's UCT value is higher or equal to the urgency value, that action is chosen. Otherwise, the first unexplored action is chosen instead. The idea is to have the MCTS fringe simulated better than in a regular algorithm.

The enhancements of* Early Cutoff* and* Unexplored Action Urgency*, introduced in [18], were further included in the PhD thesis [19]. This thesis is also a rich source of information about CadiaPlayer.

### 2.3. Simulation Control Enhancements

Most of the original contributions to the MCTS can be divided into three categories based on the area where they are applied: selection step, simulation step, or both. The last category was investigated by [20] by the authors of CadiaPlayer.

#### 2.3.1. RAVE

The RAVE method stands for Rapid Value Action Estimation. It was first proposed in 2007 for Go, but it was included in the CadiaPlayer's authors paper [20] for comparison and synergy purposes when it is be combined with other methods in GGP. A recent paper about RAVE which the GGP agents stem from is [21]. The aim of applying RAVE is to make the learning process faster, especially at the beginning when the tree exploration is more chaotic. In this method, every action in a tree keeps an additional RAVE value *Q*_RAVE_ which is updated every time the same action was played inside a simulation (not necessarily in the same state) and propagated up the tree like in the main method. In contrast to the main method, here many action have chance to propagate their values, not only the one which started a simulation. The obtained RAVE evaluations are linearly weighted in the UCT formula with the regular assessment as follows:

(5) $$\begin{array}{c} \beta \left(\;s\;\right)\times Q_{\text{RAVE}}\left(\;s,a\;\right)+\left(\;1-\beta \left(\;s\;\right)\;\right)\times Q\left(\;s,a\;\right),\\ \beta \left(\;s\;\right)=\sqrt{\frac{k}{3\times N\left(s\right)+k}}, \end{array}$$

where *N*(*s*) is the number of visits to a state and *k* is the equivalence parameter constant.

The RAVE enhancements increases results slightly or significantly in 8 of 9 tested games with the exception of Skirmish.

An interesting idea for an incremental improvement of the search algorithm is presented in [22]. The authors revisit the concept of Rapid Action Value Estimation which is very game-dependent in terms of efficiency. They show that with the RAVE turned on the results of games are shifted in a nearly linear way. In some games, for which RAVE is suitable, the shift is beneficial, whereas for others it is detrimental. The solution is to detect online whether it is worth using RAVE. Details of the algorithm are not included. However, the idea is to use RAVE only in subtrees of nodes where there is correlation between the non-RAVE scores and RAVE predictions. The RAVE value must fall into margin outside of which moves are considered as being too optimistic or too pessimistic.

#### 2.3.2. MAST, TO-MAST, PAST, and FAST

Four enhancements under the category of “simulation control” are investigated in the article [20] and the PhD thesis of one of the authors in [19].

The enhancements are as follows:

(i) *Move-Average Sampling Technique (MAST)*: a standard UCT plus a lookup table of actions assessment stored independently of the state they were played in during simulations. This enhancement is called History Heuristic. In [20], historically good actions bias future simulations according to the Gibbs Sampling (or Boltzmann distribution).
(ii) *Tree-Only MAST (TO-MAST)*: the same as MAST, but updates statistics only for actions within the constructed part of the UCT tree.
(iii) *Predicate-Average Sampling Technique (PAST)*: the same as MAST, but here the statistics are gathered and used not only for actions but also for the pairs 〈predicate,action〉 where predicates build the game states. The evaluation of a state is aggregated using the* max* operator over all the contained predicates.
(iv) *Features-to-Action Sampling Technique (FAST)*: a template for the most typical way of encoding cells and pieces in the GDL games is used. If it successfully detects that such objects are present in a game then the system learns the importance of particular pieces and cells using the TD(*λ*) algorithm. Then an evaluation function based on a linear weighting of features and their corresponding importance is constructed. The function is used in a similar fashion as actions in MAST (evaluates actions by their resulting states) to bias the simulation according to the* Gibbs Sampling.*

All the proposed optimizations are empirically tested with and without usage of RAVE. It is shown that various combinations provide significant benefits for certain games. The results of RAVE/MAST and RAVE/PAST were identified as the most promising ones.

#### 2.3.3. N-Grams and Last-Good Reply Policy

Another couple of enhancements to the MCTS selection phase are described in [23]. The first one is* N-grams Selection Technique* (NST) which extends MAST. The average players' rewards and the number of visits are here stored not just for actions but for longer sequences of actions called N-grams. During a game, the authors maintain sequences of lengths 1, 2, and 3 with their respective statistics. Sequence of 1 is equivalent to the regular history heuristic. Due to maintaining longer sequences, actions are evaluated in specific contexts. The statistics are used during a playout, where the simulated player checks the database of stored sequences (starting from those of length = 3) for possibility of reconstructing a particular sequence after choosing a candidate action. Actions leading to the best evaluated sequence are chosen more often. Both* Gibbs Sampling* and *ε-greedy* methods are tested. The latter outperformed the first one in the empirical experiments. Only such sequences of actions, which appear at least *k* times, affect the simulation phase to minimize randomly occurring noise. The authors chose *k* = 7 in the experiments. The second enhancement presented in [23] is* Last-Good Reply Policy* (LGRP) which had already been successful in Go and Havannah. The idea is store the best countermove for a preceding move. The best countermove is defined as resulting in the highest reward among all players. For each move, only one best-reply move is stored and every new one overwrites the existing one. The LGRP policy is used to rank the unexplored actions in the selection step and the simulation step. Both enhancements were tested independently and in combination with other enhancements using a number of games. The best players were using either NST or LGR with NST as the fallback strategy. Both enhancements improve the performance of the baseline CadiaPlayer in certain games.

#### 2.3.4. Decaying Strategies

The two mentioned simulation-control strategies, that is, N-gram Selection Technique and the Move-Average Sampling Technique, were further optimized by using them with a certain decay factor [24]. Decay is a process of decreasing importance of the older statistics with the assumption that they are more likely outdated or gathered not in the most current area of exploration in the UCT tree. The results are simply multiplied by a factor of *γ* ∈ [0,1]. Three decaying methods were investigated called* Move Decay* (after a move is made in the game),* Batch Decay* (after a fixed number of simulations), and* Simulation* (after each simulation, but only for N-grams and Last-Good Replies which occurred in the simulation). The authors also tested a combination of Move Decay with Simulation Decay. All of them improved the performance of the respective simulation-control strategies and Move Decay with *γ* =0.4 and *γ* =0.6 was the best, in overall, for the games designated in this experiment.

#### 2.3.5. Simulation Heuristics

An approach to optimizing the simulation phase by adopting various light-weight heuristics is investigated in MINI-Player [25]. The authors propose six policies, called strategies in the paper, which are used with certain probability at each step of a simulation to pick moves for players. Each simulation is driven by exactly one strategy (per player). The following simulation-based heuristics are proposed:

- *Random (R)*: the baseline MCTS policy is fast and unbiased.
- *History Heuristic (HH)*: an established enhancement to the MCTS is used here as a stand-alone simulation heuristic. Actions which are globally good (i.e., independent of a particular state) are chosen more frequently. The action-score statistics are updated after each simulation (not only those driven by HH).
- *Mobility (M)*: actions leading to states in which our player has more move options relatively to other players are favored.
- *Approximate Goal Evaluation (AGE)*: the authors propose a way of calculating a partial degree of satisfaction of a GDL goal rule. The idea is based on traversing a proof-tree, called the AND-OR tree, in a recursive manner. Two types of values, the actual degree of satisfaction and the tiebreaker, are calculated and propagated bottom-up in the tree. The formula is applied to all* goal* rules with the highest score available to each player. AGE will choose the action which leads to a state maximizing the* goal* score. The idea of AGE was inspired by FluxPlayer [26], but in [25] the realization is vastly different on both conceptual and technical levels.
- *Exploration (E)*: this strategy introduces a measure of similarity and thus difference between any two game states. First, for each action, the E strategy will look at its after-state and pick the most similar state to that after-state among the states visited before. Then, the chosen action will be the one that maximizes a difference between those two states among all available actions.
- *Statistical Symbol Counting* (SSC): this strategy relies on building a simple evaluation function during the START CLOCK. The number of facts of each type and the quantities of each symbol appearing at a certain position index in the facts are the building blocks of the evaluation function. All these quantities are tested for correlation with the game score and assigned proportional weights. Quantities which do not change are discarded. The strategy is a simplified version of a stand-alone player discussed in [27].

The strategies are evaluated online, independently for each player, in such a way that the ones which perform statistically better have a higher probability to be chosen in subsequent simulations. Three methods for heuristic evaluation were tested and the UCB algorithm was concluded to be the most suitable one.

Another contribution of [25] was a modified formula for choosing an action to play. A move is decided based on statistics gathered in* the top two levels* in the tree. The formula resembles a shallow min-max if our player has only one available move (min case) or each of the opponents has exactly one available move (max case). For the remaining cases, the quality of an action is computed by a linear interpolation, with *α* = 0.5, between its regular score *Q* and the minimal score of the action's child nodes. Actions leading to terminal states, for which there are no more nodes in the tree, have their average score *Q* multiplied by 1.01.

Although using heavier playouts results in smaller number of simulations per second, the approach has improved the baseline performance of the player performing only random simulations in 7 out of 9 games tested in [25]. Moreover, the agent equipped with the simulation heuristics achieves a higher average score across the domain of tested games.

### 2.4. Simultaneous Moves

While realization of a MCTS agent for the case of alternate-turn games is straightforward, things are getting more complicated for truly simultaneous games. In such games, the algorithm has to choose during the selection phase actions for each player and more than one player can have more than one action in a state. This can be seen as a multicriteria optimization. In addition, such games are usually much more complex due to the higher effective branching factor, which comes from multiplication of the average numbers of legal actions for each player. This problem was undertaken in [28] where the following methods were tested to deal with simultaneous moves in the MCTS/UCT:

- *Decoupled UCT (DUCT)*: each player stores separate rewards and visit counts for their tree. Actions are chosen as if there was no move joint dependency.
- *Exp3*: each player stores rewards and visit counts for their own moves but the score of each move is scaled by the probability of it having been sampled.
- *Regret Matching*: a regret matrix is maintained by each player storing cumulative regrets for playing an action instead of another one. The chosen move minimizes the regret.
- *Sequential UCT (SUCT)*: the game is virtually transformed into a sequential one where players choose actions one after another and the preceding choices are known to the subsequent players so they can respond accordingly.

The authors conclude that DUCT winning in 68% of games seems to be the safest choice but the SUCT with 63% win ratio is not far behind. Regret Matching is not performing well in general, but there is a game identified, where it outperforms other methods significantly.

### 2.5. Alternatives to UCT

#### 2.5.1. Roulette Wheel Selection

The possibility of replacing the UCT algorithm in the selection phase by a roulette wheel selection was investigated in the master thesis [29]. The roulette wheel selector is applied there in the most straightforward way. First the total score from all the average scores of actions is computed. Then each action *a*, in the order of appearance, is assigned an subinterval from [0,1] starting in the end of the previous action interval of length equal to the score of *a* divided by the total score. As an example, consider five actions from *a*1 to *a*5 with their respective intervals:

 {*a*1[0,0.05], *a*2[0.05,0.2], *a*3[0.2,0.25], *a*4[0.25,0.5],
 
*a*5[0.5,1.0]}.

Next, a number called* MoveSelector* from 0 to 1.0 is randomly generated. Finally, the first action with left-value of its interval greater than or equal to the generated* MoveSelector* is chosen.

In addition, one-move wins and losses are handled separately. A one-move is preferred over the roulette selection and in the case of one-move loss a random move is chosen instead (probably to avoid a division by zero). This alternative approach to balance the exploration versus exploitation ratio was tested only in two simple games: Tic-Tac-Toe and Nim. The resulting player was not significantly better or worse than the UCT one.

#### 2.5.2. TD-UCT

One of the recent publications [30], concerns combining the UCT score with an evaluation obtained from a Temporal Difference (TD) algorithm. Three ways of aggregation of the TD values are proposed:

(i) *TD-UCT Single Backup*: the algorithm omits bootstrapping and updates the TD values in the back-propagation phase only from the selected leaf node up to the root. There are two weighting parameters: distance to the selected leaf node and distance to the terminal state of the performed simulation.
(ii) *TD-UCT Weighted Rewards*: the simplest variant in which the TD evaluation completely replaces the *Q* evaluation in the UCT algorithm. Rewards are weighted by the number of steps to the terminal state.
(iii) *TD-UCT Merged Bootstrapping*: it is the most complex variant. It combines the TD-UCT Single Backup with fully fledged bootstrapping (updating states according to the value of the next state).

The authors chose two variants of Gomoku and three other games and report improvement of the plain UCT performance in all the tested variants. However, the first two variants do not perform well when combined with other well-known UCT enhancements such as RAVE or AMAF. The third variant, TD-UCT Merged Bootstrapping, is shown that it can be successfully combined leading to even better results.

## 3. AI-Based approaches

### 3.1. Overview

The competitive side of General Game Playing has been dominated by the Monte-Carlo Tree Search and its optimizations but it does not mean that methods having roots in the more classical AI were given up. We start our survey on this topic with a summary of achievements related to computational intelligence in GGP [31]. This work includes historical overview of four pre-GGP attempts to create multipurpose playing programs. A summary of the first three GGP competition winners is as follows: ClunePlayer [19], FluxPlayer [26], and CadiaPlayer [20]. The paper contains some remarks about possibility of adopting CI methods to GGP as well as the authors recent work of constructing a general state evaluation function. The approach is largely based on [32] and extended in [33], so we will devote a separate paragraph for it.

### 3.2. GDL and Features

When designing programs to play a specific game, one of the common aspects is to identify characteristic features of the game. Features can encode higher level properties of a state or can be building blocks which the game state is composed of. Such features help to determine whether a state is good or bad and usually are used by top players in their playing. In General Game Playing, no universal high level features exist and therefore they have to be learned online. Several articles have been published to tackle this issue. The approach is largely based on [34] how typically certain game-elements are encoded in GDL and what the features of a heuristic evaluation function derived from those GDL expression can be. The considered features are as follows:

- *Solution cardinality*: for example, if there are more than 0 elements.
- *Ordered domains*: for example, points.
- *Relative distances*: for example, capture when having the same location.
- *Distances to a fixed fluent*: for example, timeout-termination step.
- *Persistence*: for example, fluents which once become true never change.

### 3.3. Feature-Based Evaluation Functions

#### 3.3.1. Game Independent Feature Learning

**Figure 2:**
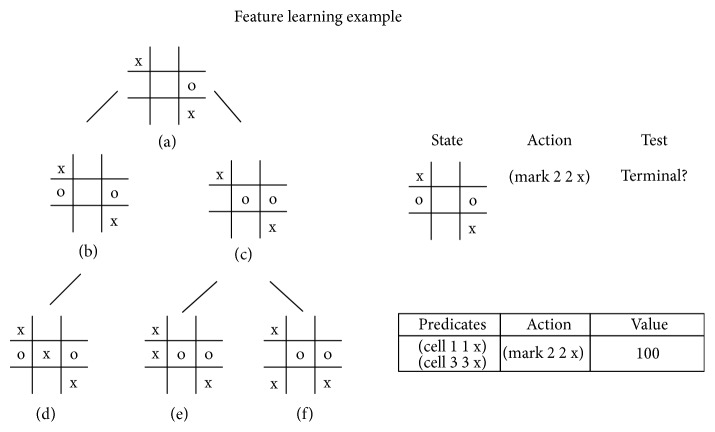
Game Independent Feature Learning. The figure was reproduced based on [35].

The paper [35] presents a robust approach to feature learning named Game Independent Feature Learning (GIFL) (Figure 2). The idea is to perform random simulations and build a small tree around a terminal state, shown in Figure 1, when the simulation ends.

Next, differences between two consecutive states encoded in GDL are extracted as a set of predicates. The features are identified as offensive and defensive depending on whether they lead to victory or prevent loss. A database of features is finally used to guide the UCT simulations. First, all applicable features are fetched based on the predicate matching with the current and the next states. Features with a value of 100 are taken immediately. If no such features exist, the applicable ones are chosen according to the probabilities computed by the Boltzmann distribution:

(6) $$\begin{array}{c} p\left(\;a\;\right)=\frac{e^{V\left(a\right)/\tau }}{\sum_{b=1}^{n}\left(e^{V\left(b\right)/\tau }\right)}, \end{array}$$

where *p*(*a*) is probability of choosing action *a*, *V*(*a*) is the value of the feature corresponding to action *a*, *n* is the number of actions, and the parameter *τ* was set to 0.5.

The approach was tested in 15 games. A significant gain was reported in 6 or 7 games depending on the time controls for moves.

#### 3.3.2. Decision Tree Learning

Identification of predicates as features is also presented in [36]. Here, the concept of a feature is simplified to a single fully grounded GDL predicate such as (cell 2 2 x). The statistics of features such as the average score, number of occurrences, mean, and high and low bound for the score are gathered during a self-play. Predicates which are more positively correlated with the win are called subgoals. In their previous work (beyond the scope of this survey), the authors were using a weighted linear combination of the features to construct a state evaluation function. Later, they switched to* Decision Tree Learning*. A Decision Tree is a widely adopted classifier in machine learning. The learning algorithm of choice was ID3. The agent performs random simulations and feeds the Decision Tree. There are some optimizations proposed to avoid creation of too many classes and overfitting. During the playout, the agent computes the next state for each available action and projects the resulting state to the decision tree. The state is decomposed into features (predicates) in order to determine which class it belongs to. The score of the class is the assessment of the state.

#### 3.3.3. General Dynamic Evaluation Function

Another approach to constructing an evaluation function dynamically was introduced in [32] and extended in [33]. The idea draws from the common and previous state-of-the-art work. Features are again predicates which are detected directly from a game description. Next, the predicates are generalized (by replacing symbols with variables) and also specialized (by replacing variables by symbols within the respective domains). Domains are detected via traversing dependency graphs of GDL fluents and variables.  Table 1 presents a possible predicate.

The features are analyzed in terms of their stability which is a function of the variance *SV* during game sequence (vertical) and variance *TV* between games (horizontal). Consider

(7) $$\begin{array}{c} S=\frac{TV}{\left(TV+10SV\right)}. \end{array}$$

A linear combination of the top 30 features by the average score is introduced. The features are weighted by a product of their stability and correlation with the game score. Such a constructed evaluation function is used in two variants: with the MTD(f) algorithm and the so-called Guided UCT method. The latter case involves early termination of the Monte-Carlo simulation with probability *p* = 0.1. In case of an early termination, the evaluation function provides scores for the players. While the approach is not yet robust enough for winning the GGP competition, in some games such as Checkers the results are very promising.

### 3.4. Distance to Features

**Figure 3:**
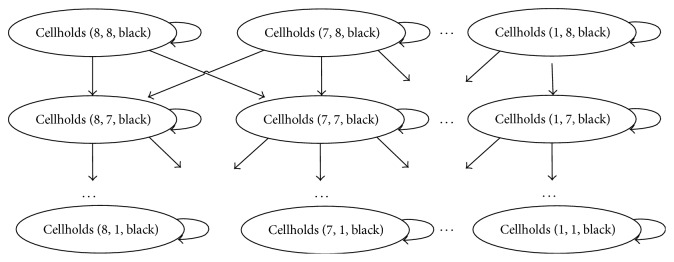
Distances between fluents in Breakthrough. The figure was reproduced based on [37].

Two papers [37, 38] investigate the concept of distance between features. In this case, the features are called GDL expression for state predicates either fully instantiated or containing variables. In earlier work, distances between two predicates required a prior recognition of board-like elements and Cartesian board-like structures with totally ordered coordinates. In the mentioned articles, the authors show a procedure for detecting admissible distance between two features by means of a number of steps required to make a certain feature true starting from a state when the other feature is true.  Figure 3 presents an excerpt from the game Breakthrough.

The method involves constructing a Fluent Graph from rules in a Disjunctive Normal Form (DNF) which is not feasible for all the games due to the rules complexity. Once distances are calculated, they are used inside a fuzzy evaluation of the degree to which goal rules are satisfied. The function operates on DNF forms of the goal rules, takes the current state as the input, and returns a numerical assessment in the [0,1] interval as the output. Conjunctions are transformed to *t*-norms and disjunctions are transformed to *s*-norms whereas* true(P)* conditions are computed based on a closest distance from the current state to the predicate *P*.

The paper identifies applicable games for which the improvement is significant. Out of 19 tested games, the method works particularly well in 3 games, slightly above average in the next 3 games, no gain is observed in 9 games, and 4 games are underperforming with the inclusion of the distance heuristics.

### 3.5. Survey-Like Papers on Knowledge-Based Methods

The dissertation [26] presents several knowledge-based methods for GGP which are as follows: (A) automatically generated state evaluation function which uses fuzzy logic to approximate degree of truth of goal conditions in nonterminal states; (B) construction of a neural network to optimize the introduced evaluation function; (C) construction of new propositions as well as solving games using automated theorem proving techniques; (D) detection of symmetry in games; (E) factoring of composite games. This PhD thesis is also an exhaustive source of information about a player named* FluxPlayer*. Because of the huge volume of this source (161 pages), we are unable to go into details like in the case of shorter articles.

Another PhD dissertation [39] presents a systematic analysis for methods for creating evaluation functions. Many overlapping concepts with [35, 37, 38] are shared. This work contains classification of approaches by a method of aggregation, performance, and the source features come from. Some theoretical divagations are included.

*Neural Networks*. In [40], the authors show how to transform a propositional logic of the GDL rules into a neural network. The rules of interest are goal rules to then perform approximation of a goal in nonterminal states. This concept has been very popular in General Game Playing. For this purpose, a generalization of the *C*-*IL* ^2^ *P* derives from the area of Neuro-Symbolic Integration (NSI). The algorithm correctly maps propositions to neurons which result in some kind of fuzzy inference engine with learning capabilities. The algorithm is described to transform rules of the form

(8) $$\begin{array}{c} q⟸\underset{1\leq i\leq k}{⨂}p_{i}\;\text{with}\;⊗\in \left\{\vee ,\wedge \right\}, \end{array}$$

where *q* is an atom and the *p*_*i*_ are literals. A rule is represented by *k* + 1 neurons where one neuron is the head of the rule and the rest *k* neurons are denoted by literals connected to the head. If the propositional value (e.g., head of the rule) is* true*, then the neuron representing the proposition responds with an output value of [*A*_MIN_, 1] whereas output of [−1, *A*_MAX_] is interpreted as* false*. In the work, a standard model of neuron is defined with real weights, bias, unbiased and biased outputs, a bipolar activation function, and the real output.

The authors tested if the mapping can be performed for the rules of 197 games. For 36 games, no network could be constructed. In 81 games, the proposed approach has led to a higher state resolution than when a straightforward, nongeneralized *C*-*IL* ^2^ *P* algorithm was used. Unfortunately, there was no GGP player built on top of this algorithm and therefore there are no results of playing strength.

### 3.6. Transfer Learning

An interesting quality attributed to human-like playing is transfer learning. It means that humans can generalize once learned knowledge about a game and use it in similar context if they appear in different games. Knowledge transfer is extremely difficult in General Game Playing, not only because the variety of games is practically unlimited but also because the description language is low-level and purely universal. Please recall that players in General Game Playing start from scratch and there is no formally provided metainformation about what game is being played or which players are involved in the game. The article [41], which uses GGP as the testing framework, concerns transfer analogy in games. The analogy is tested by comparing GDL descriptions. Two algorithms of discovering an analogy,* minimal ascension* and* metamapping*, are introduced. The first one is related to small structural changes between the descriptions (near learning) whereas the latter is responsible for matching more complex changes (far learning). Both methods apply static analysis of the GDL and dynamic analysis as the review during game play. The authors tested games within the same domain (prone to transfer) and with completely different domains. The approach has successfully identified some common scenarios, but in general, the authors conclude that there are still many limitations of the transfer learning. We will not go into details here, since transfer learning is not a part of the GGP competition protocol.

## 4. Rules Representation and Parallelization

### 4.1. Overview

In this section, we focus on dealing with the rules of GGP games and distributing computations. This includes design of inference engines for reasoning in GDL. We limit the scope to the default version (GDL-I) which has been used in all Stanford's competitions so far. In 2010, an extended specification was proposed (GDL-II) [42] which allows nondeterminism and hidden information. There are many viable ways that operate with the GDL rules such as Prolog, a custom GDL interpreter, or translation to a different representation (Table 2). A comparison between the first and the second approach is discussed in details in [43]. An overview of a few available GDL reasoners is contained in [44]. In summary, a Prolog-based engine is relatively slow.

Because the topics in this section are mostly implementation oriented, we will focus on the general report on what has been published here.

### 4.2. Instantiation

Instantiation of game description means elimination of all variables. Such descriptions can be used for many purposes such as solving games or inferring game states in a more robust form. The paper [45] comes with two techniques of instantiation: Prolog-based and a manual one using dependency graphs. A top view of the algorithm is summarized as follows:

1. Parse the GDL input.
2. Create the DNF form of the bodies and all formulas.
3. Eliminate negated atoms (in the Prolog case).
4. Instantiate all formulas.
5. Find groups of mutually exclusive atoms.
6. Remove the axioms (by applying them in topological order).
7. Generate the instantiated GDL output.

The authors were able to instantiate 96 of the 171 enabled games from the Dresden GGP repository using Prolog and 90 using the dependency graphs.

### 4.3. Propositional Net GDL Interpreter

A highly optimized custom interpreter for GDL can be created using a forward chaining technique [46, 47]. In this approach the rules are first converted to a disjunctive normal form (DNF), stratified, and ordered using a sophisticated ordering strategy based on statistics. Next, rules are assigned efficient structures which process inputs (conditions) to outputs (rule instantiations). Memory-efficient* Reference Tables* are designed for this task. The forward chaining means starting with the available ground data and traverses the rule base in an inverted fashion compared to the original GDL to get the results satisfying the required rules such as* legal* or* goal*. The predecessor article [47] is focused on the main algorithm and automatic generation of OCAML functions whereas [47] introduces later optimizations.

### 4.4. Classical GDL Interpreter

Two GDL reasoners [48, 49] approach the problem in a more classical way, that is, by determining results of rules from the definition, without full instantiation or elimination of variables, with unification of variables as they appear. Some common features of the two systems include flattening a GDL description by removing all nested arguments, compilation to *C*++, tree-like representations to perform the resolution, single-pass method by means of visiting each tree node only once, and optimized data structures for containers for the output produced by rules for conditions. For the containers, in the first approach trie-composed and tree-composed structures are used [48] whereas in the second one [49] the results are stored in memory in a linear fashion (native pointers) with dynamic hashing where it is beneficial. Other differences are in the way how the results are gathered and merged together and how unification is performed along the path of resolution or in dealing with negation and recursion.

### 4.5. Factorization and Decomposition

When having a net-based reasoning system, where each input node feeds data to an output node, it is very beneficial to decompose the graph into subgraphs to avoid unnecessary data processing. Such decomposition can be also beneficial to detect independent factors and thus reduce the complexity of the game tree. An approach to decomposition based on model checking and the Answer Set Programming (ASP) can be found [50].

Another investigated optimization is based on solving the inferential frame problem that is extracting the exact translation function from one state to another. Normally, all state predicates are cleared during the update and the GDL specifies which predicates become true after the update. A more intuitive transformation from certain predicates to certain predicates which not only illustrates how the state evolves in time but also can vastly improve the reasoning speed is presented in [51]. A related work to perform only the necessary transition from one state to another without recomputing the whole state is [52]. Like in [47], the rules are stratified and ordered. Then the so-called* numerical model of a stratified program* is constructed. Whenever a move update is performed, only the potentially affected rules are marked as “needing recomputing.” The authors report an order of magnitude improvement in Connect Four.

### 4.6. Translation of the GDL to a Different Representation

#### 4.6.1. Toss

The next concept under this category that we want to address is translation to a different representation. The GDL description can be translated to the so-called structure rewriting rules based on first-order logic with counting [53]. It allows capturing a dynamism of how predicates evolve in an automata-like graph. The method is part of the Toss system [54] which makes use of this structure to develop simple heuristics. Toss requires transformation of the GDL rules into type normal form (TNF) and like all of the players using some kind of normal form or instantiation is not suitable for too complicated game descriptions.

#### 4.6.2. Action Language

The paper [55] presents a formula of embedding GDL into an action language called* C+*. As the name implies, moves performed by players make the central point for action formalisms. The main result from the paper is the algorithm of building causal laws from GDL rules. The translation is proven to be always correct so it is a nice starting point for developing action-based heuristics.

#### 4.6.3. Planning Domain Definition Language

While in multiplayer games solving the game is possible only for trivial and let us say uninteresting cases such as Tic-Tac-Toe; in single-player games the goal is actually to solve the game at least weakly. The article [56] introduces a method to translate a given GDL description into Planning Domain Definition Language in order to use methods dedicated for planning to generate a solution to a game.

### 4.7. Parallelization

There are various reasons standing behind parallelization of General Game Playing programs such as pushing the envelope as far as possible, exploiting the parallel nature of Monte-Carlo simulations which are part of state of the art or the ultimate goal of winning the official GGP Competition. However, there have not been many articles related to parallelization in General Game Playing which is probably due to the fact that there had been already existing work tackling the parallelization in Go. To our knowledge, there are two articles [57, 58] on distributed computations strictly connected to GGP.

#### 4.7.1. Root Parallelization

In the first one, a root parallelization scheme is proposed which involves maintaining separate instances of the game tree on different machines. Statistics of nodes near the root are aggregated with certain frequency, once per move in this case. The authors investigate four techniques of such an aggregation known as* Best* (select the best evaluated move from a distributed node),* Sum* (sum of total scores and total visits),* Sum10* (*Sum* performed only for the top ten best evaluated moves), and* Raw* (send only average scores of moves from nodes without weighting by the number of total visits). The best algorithms are obtained for Sum and Sum10 and are very close. The parallelization works well for each but one tested game.

#### 4.7.2. Tree Parallelization

In the second article [58] the authors switch to Tree Parallelism, where only one master node has access to a game tree and delegates work to subplayers. The subplayers perform one or more simulations and send the result immediately. According to the article, Root Paralellism works better with small number of subplayers (less than 5) whereas Tree Parallelism scales better up to 16 distributed nodes. It becomes detrimental for a higher number of nodes in almost all tested games.

#### 4.7.3. Centurio

The last work we mention in this section is about a player named Centurio [59]. It uses the Monte-Carlo Tree Search algorithm but the realization is pretty standard, so it is not included in Section 2. Essentially, this work is a report of what is Centurio about without any novel contributions. In the case of single-player games, GDL program is translated to the ASP program in the case of solving single-player games. The authors chose an existing third-party ASP engine. Considerate part is also dedicated to parallelization on a cluster using an open-source dedicated software offering a Network-Attached Memory (NAM) implementation.

## 5. General Video Game Playing

Recently, GVGP has been proposed as a new research topic in the field of computational intelligence [60]. Although its formulation is very similar to GGP, its target has changed from traditional board games to video games. The introduction of GVGP has raised several new challenges related to the unique properties of video games. For example, video games usually do not allow very much time for players to make decisions, and this situation is exacerbated in real-time strategy games (16 to 50 ms to react). This significantly affects the possibility of using computationally expensive search techniques for GVGP. Moreover, video games typically have enemies and nonplayer characters (NPCs) that are continuously moving, and a delay in decision-making can result in significant losses within a game. Additionally, video game settings are often more closely related to real-world situations than those of board games.

Similar to GGP, algorithms applied to GVGP also need to be tested against a large number of video games. As it can be a huge burden to use many types of video games in GVGP research, the use of open game platforms is essential to increasing the speed of research. Such platforms include various video games with an API (Application Programming Interface) for the AI program. Initially, the open platforms were based on well-known emulators of early-generation console devices (Atari 2600). Because these platforms were not designed with VGDL in mind [10, 61], it was not easy to add new games to the platform, and the AI controller usually had little idea of the representation of video games on standard platforms. The most famous open game platform is ALE (Arcade Learning Environment) [62], which is based on an Atari 2600 emulator (Stella) [63]. It supports various classic Atari games, including Freeway and Ms. Pac-Man. On the other hand, GVG-AI platforms used for the IEEE Computational Intelligence in Games (CIG) 2014 GVGP competition were designed to support VGDL [11]. Moreover, the game description language (GDL) is poorly suited for video game environments because of several factors [64]:

- Nondeterministic behaviors by NPCs or elements of chance.
- Simultaneous decision making by players and NPCs at any given step of the game.
- Dynamics (physics, continuous, or temporal effects, collisions, and interactions).
- Large environments.

**Figure 4:**
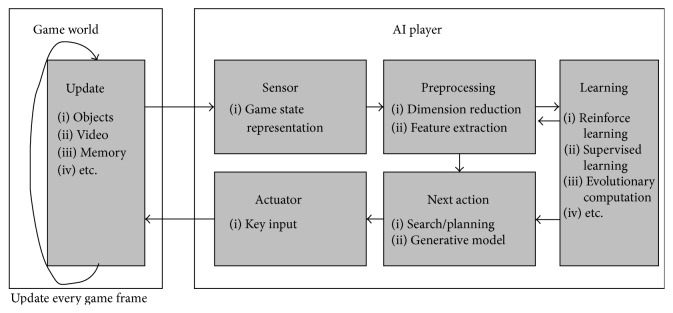
Overview of the GVGP decision process and research areas.

Although GVGP research has a relatively short history, many researchers have already applied various techniques to solve GVGP problems. These solutions have been inspired by GGP, game AI, and reinforcement learning. In this section, we will divide the GVGP problem into five subproblems: (1) search/planning algorithms, (2) learning and adaptation, (3) game state representation, (4) feature extraction and dimension reduction, and (5) objective functions. We will also discuss recent research on each of the respective subproblems. Figure 4 presents an overview of GVGP research areas and their flow of information processing.

### 5.1. Characteristics of GVGP Problems

First, most video games are played in real time. In contrast, traditional board games are based on turn-based playing, and two players typically have a few seconds to a few minutes for their turn. Naturally, AI players are also allowed to have some amount of time for the decision process. However, unlike board games, video games are based on the real-time processing of user inputs, and AI processing is accomplished between rendering frames. Although the number of frames per second (fps) varies between games, it is typically faster than 15 fps, with a 60 fps maximum, to provide gamers with seamless interactions. As a result, the AI may have only 16 to 70 ms per single frame, assuming that it can employ all of its computational resources. In a multithreading environment, the AI can work independently of the rendering engine; however, game contexts change dynamically during the AI's thinking time, forcing the decision to be quickly performed. This means time is one of the most important constraints in the context of GVGP problems. Like in GGP, MCTS has been widely adopted for GVGP; however it is characterized by a very limited number of simulations and depth, which causes a horizon effect [65].

Second, it is not easy to predict future states in video games. In traditional board games, most information is open to both players, and there are a finite number of valid moves for each piece. These characteristics make it possible to attempt to predict players' behaviors in a board game. Alternatively, video games have two significant challenges related to predicting the future outcome of the game. First, they use randomness to determine the appearance of obstacles and for NPC behavior [66]. Because the game environment is changing over time in a random fashion, it is difficult to predict future states from the current game context. Second, the number of possible actions per move is often infinite because the players are able to control units in any direction. Furthermore, similar to poker games, a part of the opponent's information may be hidden. For example, a fog-of-war is common in many real-time strategy games, and the vision of players is limited to the areas around allied units. As a result, a forward model to simulate games can suffer from inherent inaccuracy.

Finally, the general definition and acquisition of relevant features for GVGP are not trivial because each game has a variety of different game objects, whereas the observation of objects can change with the viewpoint settings. In board games, the game space is bound to the board and all the information on the board is open to both players. In video games, there are many different types of game objects including, but not limited to, animals, monsters, items, and natural objects. It is not easy to convert the game objects in each game scene into vectors of numbers for evaluation functions. Additionally, there are many different techniques to support the generation of diverse views of game scenes. For example, there can be a third-person perspective, zooming in and out, and so on. Moreover, in some video games (in particular those protected by commercial laws), the acquisition of gaming events is not allowed. In this case, researchers often use screen-capture-based image processing and direct memory data access to extract information from scenes.

### 5.2. GVGP Platforms

Because GVGP is a relatively new research topic, there are a small number of available platforms for benchmarking purposes. The oldest platform is ALE (Arcade Learning Environment), which was proposed by the Alberta games group and uses Atari 2600 games [62]. Recently, D. Perez et al. developed a GVG-AI platform inspired by ALE, and this platform was used at the GVGP competition held at the IEEE CIG conference [11]. In addition, there are open-source platforms such as Learnfun & Playfun (L&P) [67] and Piglet [68] that use software emulators. Although these two platforms have yet to be described in the literature, their design is similar to that of ALE.

#### 5.2.1. Arcade Learning Environment

**Figure 5:**
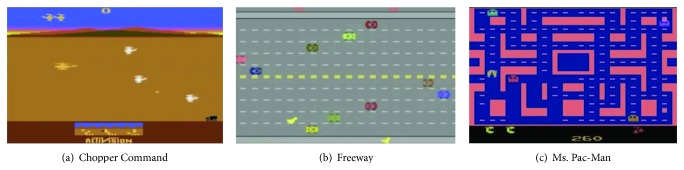
Examples of games in the Arcade Learning Environment.

ALE is an open GVGP platform developed by the Atari 2600 game group in Alberta (http://www.arcadelearningenvironment.org/). It is based on Stellar, an Atari 2600 emulator, and, most importantly, AI programs can be developed for this platform. Atari 2600 was a home video game console released in 1977 and has more than 500 available games. Among these are traditional games such as Pac-Man and Space Invaders (Figure 5). The main advantage of the platform is that it supports older games released in the early days of the video game industry. Due to the variety of supported games, ALE is a good platform for GVGP research. In addition, games can be simulated by storing the emulator's memory, registers, and states. For these reasons, ALE has been used extensively in GVGP and reinforcement learning research [62, 69–72] and can be used for search/planning with MCTS and model-based reinforcement learning. Additionally, the system in this platform receives 18 action inputs by emulating a joystick with one push button. Finally, the AI developer can have video outputs and memory states from the emulator.

Because the ALE platform is based on an emulator, there are some restrictions on the interface between the AI and the console game. Although, there is no restriction on emulating the inputs, the gaming events themselves must be interpreted from the raw data, including visual output and memory data. Image processing (or vision) algorithms can be applied to a captured two-dimensional screen image to identify objects and backgrounds. Because this processing takes time, sophisticated vision algorithms cannot be used to enhance the accuracy of recognition. As a result, the game-state data from a console game is uncertain and is of low resolution with less structured forms.

#### 5.2.2. GVG-AI Competition Platform

The GVG-AI platform was developed to promote ALE-inspired GVGP. ALE supports diverse games from Atari 2600, and there have been successful research initiatives using the platform. However, successful ALE-based GVGP research is hindered by the difficulty in extracting game-state data from the raw data. This was an inevitable problem for the ALE platform because it was based on game emulation. Although performing GVGP with raw data is similar to human-like game play, it significantly increases the problem complexity. On the other hand, the GVG-AI platform can collect information on the current game state, such as object instances and validity checks of actions. Taken together, this allows for game simulation based on forward models. In this way, this platform avoids the technical problems of emulator-based systems and allows researchers to focus primarily on solving GVGP issues.

In contrast to ALE, GVG-AI utilizes VGDL, which is similar to the GDL used in GGP [10, 61]. In GGP, GDL is used to define game rules. Similarly, VGDL can define the rules of two-dimensional video games. Each VGDL description can be translated into a game in the GVG-AI platform. Inside the platform, a VGDL description contains the game logic required to run the game and all the computational resources are available to the AI developer, regardless of the VGDL definition. Specifically, the GVG-AI platform uses a VGDL JAVA programming language port of the initial PyVGDL format that was designed as a subset of Python.

**Figure 6:**
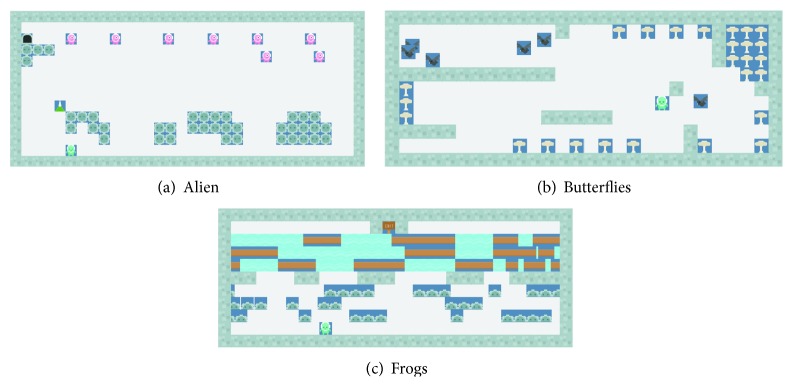
Examples of types of games in the GVG-AI competition platform.

The limitations of the GVG-AI platform are that it supports a limited number of games and that there are some restrictions on the creation of games. Although it is easy to define video games using VGDL, there were only 20 such games available at the 2014 IEEE CIG competition. Relative to ALE, this is a small number of games that does not cover the plethora of game genres (Figure 6). To overcome these shortcomings, there have been efforts to automate game-level generation using PuzzleScript, which is similar to VGDL [73]. Because VGDL is designed to automatically create games and generate procedural content, it is expected that the GVG-AI platform will have more games in the near future [74]. At this moment, however, VGDL supports only a two-dimensional grid-style game environment, which hinders the creation of new types of games. Recent projects have focused on extending the original VGDL to first-person shooting games [10].

#### 5.2.3. Learnfun & Playfun and Piglet

There are two additional platforms, L&P [67] and Piglet [68], which were developed independently. However, they were not developed specifically for GVGP or game AI research and are not well-represented at academic conferences or in journals (although L&P is partially described in [75]). Instead, these two platforms have been posted on personal blogs, GitHub, and YouTube. The authors have made the platforms available for GVGP research, although there is little connection between them and the foci of academic GVGP research.

**Figure 7:**
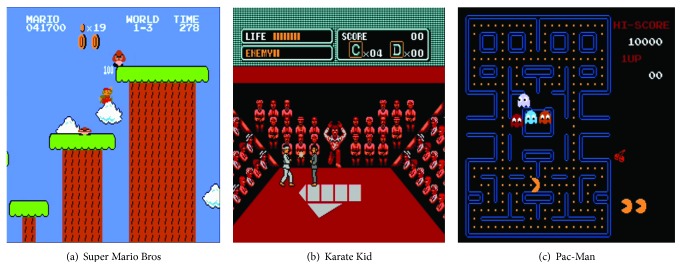
Examples of games available in the Learnfun & Playfun platform.

Learnfun & Playfun was implemented using a Nintendo Entertainment System (NES) emulator. The primary goal of the creator was to design software to play games without human intervention. As a result, L&P was designed to learn how to play a game using game-play data without the need for game-specific knowledge. Although this learning process is time-intensive, the trained AI can eventually successfully play many different types of games. Additionally, L&P automatically determines which variables are consistently increasing in the memory of the learned data and uses this as a target function. For instance, although software may be designed for Super Mario Bros, it is possible that it can also play other games, including Hudson's Adventure Island, Pac-Man, Karate Kid, and Bubble Bobble without significant changes in the code (Figure 7).

**Figure 8:**
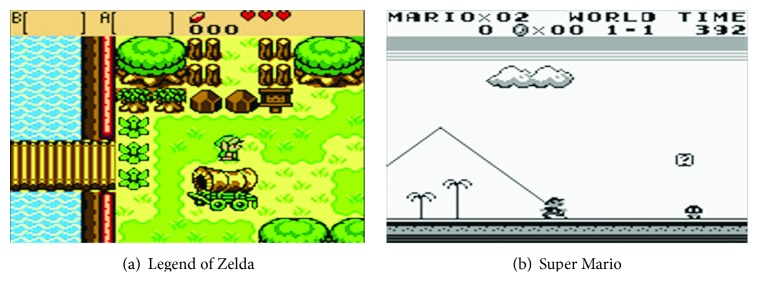
Examples of games available in the Piglet platform.

Piglet, which is similar to L&P, is based on a Gameboy emulator (Figure 8). Its author noted that he referred to the L&P work while developing Piglet. The main difference is that the target function in Piglet is based on curiosity and novelty. Although L&P was designed to increase numbers corresponding to item counts, scores, and positions to rank variables for the target function, Piglet plays games in such a way as to maximize the number of changes in the memory. This curiosity-based approach (seeking novelty) has been used to solve deceptive problems [76–79].

### 5.3. Algorithms for GVGP

In video game AI, the controller can be seen as an agent with sensors and actuators [65]. It continuously collects data from the game environment using logical sensors and makes decisions for actuators. Although many techniques have been developed for game AI, they are usually coupled to predetermined target games, making them more or less game-specific. Because video game AI is highly dependent on game-specific knowledge, it does not generalize well to other games without major revisions. This means that successful techniques used in conventional video game AI design are likely to fail when used for GVGP without consideration of “generalization.” As in GGP, it is desirable to use domain-free knowledge (less dependent on game-specific knowledge) to train algorithms capable of automatic game analysis. From this perspective, we can divide the GVGP problem into three subparts. In this section, we will describe each subproblem with related works:

- Feature extraction and dimension-reduction techniques to improve learning efficiency.
- Search/planning algorithms independent of any domain knowledge.
- Efficient learning algorithms that can learn new environments.

#### 5.3.1. Inputs and Feature Extraction/Reduction

There are different types of inputs available in the GVGP platform. In the GVG-AI platform, inputs are represented as structured game objects [80]. These provide an API to the information on gaming objects, with some restrictions in competition mode. In this platform, the AI can determine the position, speed, number of items, number of enemies, and player position if allowed by the API. Unlike the GVG-AI platform, the ALE platform is based on emulators and therefore provides strikingly different inputs: a computer screen image (color values of pixels) and memory data. Although this is more like human visual processing by way of raw-level inputs [81], it significantly increases the complexity of preprocessing. The computer screen image input requires a vision algorithm to segment the gaming object from backgrounds and tricks to identify the different types of objects (e.g., mines or enemies) [82]. In addition to the screen inputs, raw memory data from emulators can be used as an input because they contain different types of information about the current game state. One way to evaluate the informative memory is to use the lexicographic order [67].

The size of the inputs in ALE (i.e., screen images and memory) is relatively large: the screen resolution is 160 × 210 (33,600 pixels) and the memory has 1024 bits. Because the raw data is so large, the detection of game objects in this search space is not a trivial problem. For example, to locate the player avatar, feature sets are generated by exhaustively enumerating all single patterns of sizes 1 × 1, 2 × 2, and 3 × 3 [69]. This produces 569,856,000 different feature sets. To address this complexity problem, researchers have used a tug-of-war hashing algorithm to reduce the dimensions of the input data. Alternatively, a different study [70] proposed that the “contingency awareness” concept drawn from cognitive science to be applied to GVGP. The contingency awareness concept rests on the premise that the ability to know some aspects of future observation is under the agent's control, whereas other aspects are determined solely by the environment. This is important in GVGP because it helps identify important areas of interest, which are defined as contingency revisions by the authors. This approach was tested using 46 games in ALE and involved segmenting the input space into several regions, thereby reducing the amount of information processing. Similarly, in [71], a large observational space was decomposed into a number of smaller, more manageable subproblems by factoring the raw inputs using 20 different Atari 2600 games. Recently, Mnih et al. [72] showed promising results for ALE platform games using deep learning techniques and the automatic feature extraction of high-dimensional inputs.

#### 5.3.2. Search/Planning Methods

Similar to GGP, MCTS is one of the most promising techniques in GVGP research. The GVGP competition, hosted at IEEE CIG 2014, showed that MCTS is also one of the most popular techniques in GVGP. In [62], the authors reported that MCTS performed significantly better than a breadth-first search on the ALE platform. It is not an easy task to achieve good performance without domain-specific knowledge. Perez et al. proposed using a knowledge-based (KB) enhancement of MCTS called KB Fast-Evo MCTS, which takes advantage of past experiences [80]. They reported that the use of a KB significantly improved the performance of the algorithm on the GVG-AI platform. Alternatively, Vafadost developed techniques for temporal abstraction inside MCTS for the effective construction of medium/long-term plans on the ALE platform [83]. In this study, the Variable Time Scale (VTS) was shown to be a promising technique for determining the optimal time scale for taking each action.

There are several problems with the use of MCTS in GVGP. The main difficulty is the limitation on the number of simulations per frame. In the GVG-AI framework, the controller has approximately 40 ms between each frame. Because the game state is updated at each frame, it is not practical to wait for multiple time frames to engage in long-term planning, as it is necessary to respond with one action per frame. Additionally, waiting increases the inaccuracy of simulations because the game state is likely changing while the simulation is running. Another difficulty when applying MCTS to the GVG-AI framework is the randomness of NPCs and enemies. Because the MCTS algorithm is based on a large number of simulations derived from the current game state, it is essential to have an accurate prediction of future game states after a finite number of actions. However, there is no way to accurately predict the future positions of game objects in video games with regard to randomness (e.g., related to NPCs and movement). As a result, MCTS must use uncertain predictions for future game states. Finally, the search space of games is usually extremely high. In traditional board games, the number of possible game states after a finite number of actions is determined by the possible valid actions per each player move (defined as a branching factor). Although the GVGP platform is based on two-dimensional games, which have a limited number of valid actions per time frame, the game states are also being affected by the positions and states of NPCs and enemies. Moreover, the number of movable gaming objects per frame is not small (as shown in Figure 6), and it compounds to cause an exponential growth of possible states.

Although MCTS has been the most successfully applied approach in GVGP research, evolutionary algorithms have also been used to tackle the problem. For example, the IEEE CIG 2014 GVGP competition featured an MCTS as well as a simple genetic algorithm (GA) approach. The final rankings showed that although the GA approach was not as competitive as MCTS, the GA showed potential. Based on this result, it is predicted that hybridizing an evolutionary algorithm with MCTS (e.g., KB Fast-Evo MCTS) will allow a synergy between the two search techniques [80].

#### 5.3.3. Learning and Adaptation Methods

In learning methods, the AI controller attempts to learn how to play the game. In [62, 69], SARSA(*λ*), a traditional technique for model-free reinforcement learning, was augmented with a linear function approximation. The parameters of the learning algorithm were tuned by training on five games and then tested on 50 games. The goal of the agent was to maximize the accumulated award by observing action-reward loops. The authors reported that the learning approach showed potential on the ALE platform, but there was room for performance improvement. Additionally, during the learning process, different types of feature representation methods were compared using Atari 2006's screen and memory. The conclusion was that there is no dominant learning method that can cover all the games. Recently, Mnih et al. proposed the use of deep neural networks for reinforcement learning problems on the ALE platform [72].

In a model-based approach, the goal of learning is to find a model that properly selects the next action based on the current game state. Recently, an evolutionary artificial neural network developed for GVGP incorporated a large number of neurons and connections [82, 84]. In [82], the authors found that Hypercube-based NeuroEvolution of Augmenting Topologies (HyperNEAT; an evolutionary neural network), which can handle high-dimensional inputs, was promising for two games on the ALE platform. The authors preprocessed game screens using image-processing techniques to generate inputs for the neural networks, which, in turn, returned key actions. In [84], the authors showed comprehensive experimental results of different types of evolutionary neural networks (or NeuroEvolution, NE). They compared Conventional NE, Covariance Matrix Adaptation Evolution Strategy (CMA-ES) NE, Evolutionary of Network Topology and Weights (NEAT), and HyperNEAT on 61 test games. They found that direct encoding algorithms (such as NEAT) outperformed the other methods on low-dimensional, preprocessed objects, and noise-screen representations but the indirect encoding method, HyperNEAT, was promising for fully generalized raw-pixel representations.

### 5.4. Relationships between GGP and GVGP

Technically speaking, GDL could be used for video games. It allows nondeterminism (in the GDL-II variant), simultaneous actions, and any number of players. However, it was not tailored for the application in video games and there are better solutions for this task.

The primary reasons of why GDL is not used in GVGP are as follows:

- Complexity of defining sophisticated video games, especially real-time ones with continuous (frequently occurring) events: such definitions would end up in GDL being very bloated and unnatural. They would typically require extensive amounts of rules, lots of artificial timer facts which are counterintuitive and difficult to read by humans, and many state updates only with no-op (no operation) moves from players because, in video games, states often change regardless of the player's actions. Such complex and extremely lengthy descriptions are difficult to maintain or even understand by humans.
- Complexity of simulating video games in GDL: the GDL interpreters are relatively slow. Video games typically require fast responses from the players (e.g., 40 ms). Given the fact that descriptions in GDL would already extremely be complex in the case of real-time video games, the available time could be even too low to carry out a single simulation of a video game in GDL. There are no prospects that this could change in any foreseeable future.

The distinction between GGP and GVGP is done already in formulation of goals in both frameworks. They were designed to work in parallel, rather than interfere or extend each other. GGP focuses on combinatorial mind games which may have complex rules as long as they retain the discrete nature. In such games, a state is updated much less frequently than in GVGP and usually as a direct result of the actions taken by players. GVGP games may even have simpler structure, but the setup is more complex as the games are played with a much faster pace and events causing the state update may occur at any time. Therefore, GVGP needs a dedicated framework optimized for this kind of usage.

Thanks to the optimal usage of GDL and GVGL, the respective competitions (GGP and GVGP) may operate using a vastly different set of games which differ in the properties mentioned in the previous paragraph:

- GGP: mostly discrete combinatorial nature, the game state usually changing to players' actions, and typically 10+ seconds for a move
- GVGP: mostly continuous nature, frequent incremental changes of the game state, and typically 40+ ms for a move

Although two games, which can be considered video ones, that is, Pac-Man and Street Fighter, have been defined in GDL, they were significantly simplified compared to their original counterparts.

## 6. Benchmarking and Competitions

In GGP, the official competition [7, 85] plays an important role in encouraging and promoting research; naturally, it is used for benchmarking. Since 2005, the competition has been associated and colocated with either the Association for the Advancement of Artificial Intelligence (AAAI) or the International Joint Conference on Artificial Intelligence (IJCAI). The number of participants is usually between 10 and 18. The tournament consists of two phases. The preliminary phase is open to all participants, and the top eight teams advance to the finals (except for 2014, when the top 12 advanced). In the finals, the best player is chosen in a double-elimination playoff format. At each phase, different types of games are used. For example, the preliminary phase may use single-agent, two-player, and multiplayer games. However, for the finals, only two-player games have been used so far. In the final stage, each match between two players is played in a best-of-three setting. The competition includes turn-based or simultaneous move and zero-sum or non-zero-sum games, including anything from simple puzzles to complex chess-like games. Some variants of popular board games have been used, such as checkers played on a cylindrical board or Tic-Tac-Toe played in parallel on multiple boards.

The GGP competition has shown steady progress in the performance of the strongest program and has evolved to incorporate human versus machine matches (known as carbon versus silicon) after the official tournament phase. Except for the first year of the event, the machines have outperformed human players. After several years of competition, progress has become apparent as the new players can easily beat the old players. There are several sophisticated approaches implemented in the GGP competition, such as game-independent heuristics (mobility, inverse mobility, and goal proximity), learning weights on game-playing heuristics, MCTS, and structural analysis and compilation [85]. In 2013, a General Game Playing course was offered online by COURSERA [86], which has led to a significant increase in competition participants.

The success of the annual GGP competition has inspired the GVGP community to start a similar event in 2014 [11]. The GVGP competition is based on the VGDL and provides 10 sample games for training purposes. An additional 10 games each are used for validation and testing stages. However, these 20 games are not open to the public until the day of competition. Participants are allowed to test their algorithms using the 10 validation games to obtain scores, but the VGDL files themselves are not available. The VGDL is designed for modeling two-dimensional grid environments with the protagonist, nonplayer characters, obstacles, and other objects; Pac-Man and Space Invaders are examples of games that can be modeled by VGDL. The VGDL consists of the following components:

- Sprite Set: all available sprites for the games (parameters and display settings are also included).
- Level Mapping: relationships between characters and sprites.
- Interaction Set: specification of events when two sprites collide in the game.
- Termination Set: end condition of the game.

Because the VGDL is not visible to the AI in the validation and testing stage, it is not easy to initially understand the goal and objectives of a game. As game play progresses, the AI controller must determine the goal of the game, how to increase the controller's scores, the events that occur when two sprites collide, and the nature of the sprites in the game. The organizers provide four different types of example controllers: random, one-step look ahead, genetic algorithm, and MCTS. Because they are allowed only about 40 ms per frame, it is important to efficiently simulate future game states.

In the 2014 competition, 14 participants submitted their entries. For comparison, the four example controllers were included in the evaluation. The best AI player was ranked first in five in ten hidden test games. Based on the description of the winner, it used “an open loop” tree search to build a tree representing sequences of actions. The open loop means that no state is stored in the tree. The UCB (Upper Confidence Bounds) formula introduced a “taboo bias” penalty for actions leading to avatar positions visited in the recent past. Also in the 2014 competition, the two best players outperformed the sample MCTS.  Table 3 summarizes the players, scores, ranks, and the techniques used at the IEEE CIG 2014 competition.

To evaluate AI, organizers executed 10 games with five different levels. In total, this yielded 500 games per AI (500 = 5 × 10 × 10). To rank the AIs, the organizers used the (1) number of victories, (2) total points, and (3) elapsed time to compete levels. The number of victories was the most important factor, but the total number of points was used as a tiebreaker in cases of a draw. Each AI received a point score based on its ranking: the first-place entry received 25 points, the second-place entry received 18 points, and the third-place entry received 15 points; the entries in lower places received 12, 10, 8, 6, 4, and 2 points and 1 point, respectively. Entries ranked lower than 10th received zero points.

## 7. Challenges

Despite significant development of GGP and GVGP domains in recent years there are still many open questions and challenging issues which are worth considering and interesting research topics. Some of them, chosen based on the subjective preferences of the authors, are listed below.

*Human-Like Playing*. Cognitively-plausible and human-like playing [3] has been a challenge in AI which is still unsolved. A majority of the top players use the MCTS algorithm which can be considered a refined brute-force approach relying on a high number of simulations. The MCTS-based players tend to play many games in a similar way which can be spotted and exploited by a human player. Extensive calculations are rarely involved in human-like playing. Instead, we rely on intuition, creativity, experience, and detecting visual patterns while playing [3, 5]. Bringing all these four concepts to GGP is a grand challenge. Tackling intuition could be started from a robust method of focusing the machine (simulations) only on certain actions and discarding unpromising ones very quickly. Detection of visual patterns could be started off by a method of an automatic visualization of game states in GGP.

*Opponent Modeling*. Opponent modeling is an important asset of game-playing programs and the realization of this concept poses a challenge in many games. Naturally, a proper opponent modeling is crucial to games in which there are many iterations of the same game against the same opponents such as Poker [87]. But it is more than that; the tree search algorithms quietly assume some kind of (usually rational) opponent's behavior. Having a proper opponents' model, the GGP agents could prioritize exploration of certain parts in the game tree associated with actions more likely to be played by the opponents and have the better assessment of these states. Knowing a profile of an opponent could also alter the strategy of our player. So far, there had been only a limited success in implementing this concept in GGP.

*Game Description and Representation*. The use of GDL as a game-defining framework makes certain approaches prohibitive. Firstly, simulations of games written in GDL are slow because of several reasons. Some constructions such as math expressions including very basic arithmetic, ordered data types, and loops are not part of the language and have to be simulated implicitly. Secondly, a suboptimal performance of GDL is a price to pay for its universality. Because of this universality, a GDL description contains no information about the game except for the way of computing the initial state, legal moves, state updates, and verification of whether the state is terminal and if so what are the goal values for the players in that state. There are no clues about what kind of objects constitute the state or what does a particular fact mean. Additionally, there is no way to put any metadata in the GLD game description. Furthermore, while a GDL game description can be easily used to formal simulation of a game, it is almost impossible, in a general case, to detect what the game is about and which are its crucial, underpinning concepts. On the other hand, a majority of successful game-dedicated programs (e.g., in chess, bridge, or go) dwell on the game-related concepts.

We would like to pose three challenges in this area:

- To replace GDL by a more game-oriented (while still general) description language.
- To come up with an automatic way of translating rules written in GDL to a more efficient representation in terms of performance and access to knowledge.
- To design a method of discovering game-related objects in a game written in GDL in order to build the internal game representation.

*Transfer Learning*. Transfer learning means reusing knowledge learned while playing one game in playing another game. In GGP, this concept can be tackled in the following three ways:

- Changing the GGP specification to include a unique name in the GDL description: in this way, transfer learning between matches of the same game could be naturally implemented.
- Performing automatic mapping of equivalent games (even if the descriptions are obfuscated, the order of rules is changed, etc.): such idea was pursued in [88] by means of automatic domain mapping from a formal description of a game, which was consequently used to transfer an evaluation function between games.
- Retaining the GGP specification but still enable transfer learning: to this end it would be necessary to extract universal high-level concepts existing in many games (to enable transfer) and design algorithms which operate on them (to enable learning and usage).

Transfer learning can speed up the learning process as players would not have to learn from scratch. It could also reveal insights about similarities between games which would be especially useful for game descriptions related to or inspired by real-world problems. The concept of transfer learning clearly overlaps with human-like playing as humans intuitively transfer game-playing experience between similar games.

*The Use of Computational Intelligence*. Computational Intelligence [89] encompasses variety of methods which are adaptive and general and can be used in games without any* a priori* knowledge. Among them, most notably, neural networks and a rich family of metaheuristics seem to be a perfect choice for multigame playing. However, CI-based methods have not yet had much success in GGP. The achievements and perspectives as of 2010 can be found in [90]. Probable reasons why the CI algorithms are not fuelling the state-of-the-art players are the limitations of GDL and relatively short time available for learning in the GGP protocol. On a general note, CI-based learning methods are often too slow even with specialized game engines. The lack of game-related features present in GDL also hampers application of many CI methods. Such features could, in principle, be used as an input to a neural network or expressed in the form of genomes in evolutionary approaches.

While GDL shortcomings generally hinder the efficient use of CI methods in competitive GGP, we believe that multigame playing (in the form of GGP or another) is, nonetheless, one of the grand challenges for CI/AI [3, 5, 31].

*Technical Challenges*. Improving the official General Game Playing Competition has been a constant challenge for the organizers. The community needs new games, higher number of unbiased games (with equal chances of winning for all players), a better communication protocol (perhaps not requiring participants to host their players as they were servers), and finally a way to attract more participants.

The MCTS algorithm used in GGP could be improved further. In particular, the algorithm could be tweaked online to better suit the currently played game and also use some knowledge discovered in this game. Better parallelization schemes, preferably adjusted online to the played game, and faster inference engines are relevant technical challenges as well.

*Further Investigation on Video Game GGP*. Recently, the GGP for video games has been newly introduced to the computational intelligence and games society. It is similar to traditional GGP research on board games but includes video games, more challenging and close to real-world commercial games. Because it is quite new research area, there is very small number of publications available so far compared to GGP. However, there are very interesting fundamental building blocks for future successful research including GVGP platforms, VGDL, and GVGP competitions. The next problem is to define more useful GVGP platforms, extension of VGDL for complex video games, automatic creation of video games using VGDL, cross-fertilization between GGP and GVGP research, and application to commercial product.

## 8. Summary and Conclusions

In this survey, we have listed recent advances in GGP since 2011. Although we cover just four years, there has been big progress related to MCTS, GVGP, and competitions in this time frame. In the GGP research, there have been successful papers on hybridization of game-independent search/planning/heuristics and knowledge extraction from game playing. The boundary of GGP has been expanded to video games by the introduction of emulator-based platforms (e.g., ALE) and VGDL-based platforms (e.g., GVG-AI). The introduction of GGP to video games raised several new challenges: what are the important inputs from video games (memory or screen visual inputs)? Which GGP techniques remain successful for GVGP? A new challenge is also related to the invention of VGDL and a variant of GGP competition dedicated to video games (GVGP). Successful approaches to GVGP revealed important new insight on the understanding of GGP for video games. They can give useful advancement in engineering and ideas for cognitive science to understand human's generalization ability when playing games.

Compared to the GGP competition, which was initiated in 2005, the GVGP competition is at quite an early stage of development (it is just one year old as of 2014). The competition organizers have not yet created enough new video games and, therefore, the expression of the VGDL is still limited. Also, there are not much education resources for GVGP competition as opposed to the GGP competition. For the latter, there is a massively online open course (MOOC) available. Nevertheless, the progress in GVGP field is clearly visible: a new game description language (VGDL) was specified and several platforms, hobby-style research works, and media exposure (deep mind [72] acquired by Google company) appeared recently. Lately, a group of leading researchers in GVGP had a meeting at Dagstuhl in Germany [9]. We believe that the field will soon become one of the most important areas of the game AI research.

## Figures and Tables

**Algorithm 1:**
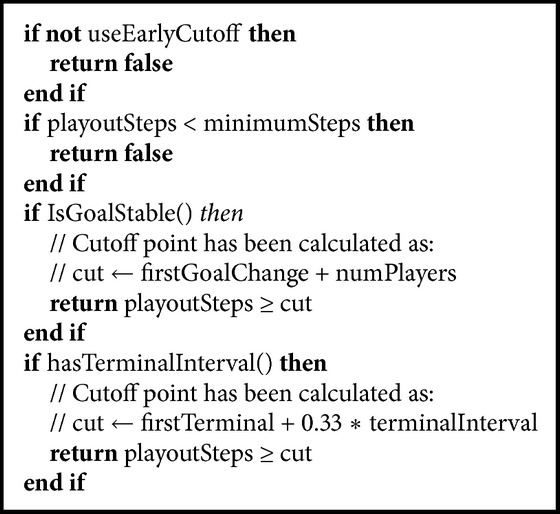
Pseudo-code for deciding cuts for the early cutoff extension. It was taken from [18].

**Table 1:** 

Example predicate from the rules	New predicates after generalization	New predicates after specialization
( cell 1 1 ?)	(cell ? ? ?) (cell 1 ? ?) (cell ? 1 ?)	(cell 1 1 x) (cell 1 1 o) (cell 1 1 b)

**Table 2:** 

Approach	Feasibility	Speed
Full instantiation of all states	*⋆*	*⋆⋆⋆⋆⋆*
Propositional network	*⋆⋆⋆*	*⋆⋆⋆⋆*
Custom GDL interpreter	*⋆⋆⋆⋆⋆*	*⋆⋆⋆*
Prolog interpreter	*⋆⋆⋆⋆⋆*	*⋆⋆*
GGP Base Java Package	*⋆⋆⋆⋆⋆*	*⋆*
Another representation	?	?

**Table 3:** Summary of entries in the IEEE CIG 2014 competition.

Rank	Entry name	Total score	Approach
1	Adrienctx	158	(i) Open loop tree search with UCB (ii) Taboo bias
2	JinJerry	148	(i) Multistep look forward (ii) Heuristics
3	sampleMCTS (sample)	99	MCTS
4	Shmokin	77	(i) MCTS (ii) Hill climbing
5	Normal_MCTS	68	MCTS
6	Culim	61	Online Q-learning
7	MMbot	59	MCTS
8	TESTGAG	49	GA (Genetic Algorithm)
9	Yraid	49	RBS (Rule based System) with GA
10	T2Tompson	47	(i) Steepest-ascent Hill Climbing (ii) Random move (iii) Horizon capped A*∗* search
11	MnMCTS	47	MCTS
12	sampleGA (sample)	43	GA
13	IdealStandard	39	(i) Find all nonlethal sprites by simulation (ii) Visit nonlethal sprite randomly
14	random (sample)	35	Random move
15	Tichau	30	
16	Sampleonesteplookahead (sample)	17	One step look ahead
17	levis501	11	Multistep look ahead
18	LCU_14	4	
